# American Medical Association

**Published:** 1871-05

**Authors:** 


### Miscellaneous.

### American Medical Association.

### FIRST DAY.

The twenty-second Convention of the American Medical Association was opened in Pacific Hall, San Francisco, Cal., on Tuesday morning May 2d, at 11 o'clock. The present officers are: President, Dr. Alfred Stille, of Pennsylvania; Vice-Presidents, Dr. J. S- Wetherby, of Alabama, Dr. Henry Gibbons, of California, Dr. G. J. Heard, of Texas, Dr. Samuel Willey, of Minnesota. Permanent Secretary, Dr. W. B. Atkinson, Philadelphia; Assistant Secretary, Dr. Joseph Tucker, of California; Treasurer, Dr. Casper Wistar of Pennsylvania: Librarian, Dr. F. A. Ashford, of District of Columbia.

Dr. Arthur Stout, of San Francisco, called the meeting to order, and introduced the President of the Association, Dr. Alfred Stille.

President Stille received a warm greeting from the meeting. He introduced the Right Rev. Bishop Kip, of California, who invoked Divine blessing upon the proceedings of the convention.

The report of the Committee upon Credentials was called for. Dr. Stout, the Chairman, delivered an address in which he heartily welcomed the members to the hospitalities of California.

Dr. Stout reported that the registration had not yet been completed, two hundred members having thus far been registered.

On motion, the Committre were given until Wednesday to present their report.

A letter was read from Prof. S. D. Gross, of Philadelphia, ex-President of the Association, regretting his inability to attend the sessions of the Convention. It was ordered spread on the minutes.

On motion of Dr. Stout, all members of the California State Medical Society not delegates were invited to sit as members by invitation.

The President commenced his annual address by calling attention to the vast change which had taken place in the State of California during the quarter of a century of the existence of this Society. He then adverted to the objects for which the Association was formed, and the progress which had been made in the profession, as he felt, by its agency. Further maturity, however, he said, was needed, a higher growth was to be looked for; the idea of developement in education is as natural and as necessary as it is in the growth of an organized being. In speaking of advance in professional education, he considered it a fact that, although scarcely one of the many reforms recommended by the Association had been formally adopted by the colleges, medical education has been continually improving. Obstacles to farther and more rapid improvement exist and must be met.

"Either some one institution must be endowed so as to be rendered independent of its rivals, or a number of the leading schools must agree together to adopt a curriculum in harmony with the present state of medicine, and with the system of instruction pursued in the principal schools of the world. Of these two conditions there seems no prospect whatever that the first ban be fulfilled. The execution of the second depends entirely on the good will of the colleges that are interested in the decision. No one can act alone ; and every efiort to induce several of them to enter into a compact which shall be of mutual obligation, and not to be abrogated without the consent of all contracting parties, or, at least, a large majority of them, has hitherto proved unavailing. What motives, if any, will determine the adoption of a different policy, may be conjectured, but need not be suggested ; yet it is safe to affirm that if the profession at large were to lend their support to those colleges and only those which determine to carry out essentially the recommendations of the conventions of medical teachers held at Cincinnati in 1867, and at Washington in 1870, we should soon enjoy tne benefits of a system of education which would place the American medical profession upon a perfect equality with that of the most favored country."

Dr. Stille spoke, in sequence, of quackery, of the question of women entering the profession, of colored physicians, of the granting of diplomas, of the right of colleges to revoke the diplomas of men who leave the ranks ot legitimate medicine for quackery, and of alcoholic stimulants as medicines.

At the conclusion of the address a vote of thanks was accorded to the President.

Several invitations of an agreeable nature were extended to the members of the Association, which were accepted.

The reports of a large number of Committees were expected. But few of them responded to the invitation of the chair, and those principally to gain time. The report "On Protest of Naval Surgeons, &c., by Dr. S. W. Ruschenberger, U. S. N., was read and was laid on the table. That "On a National Medical School," by Dr. Francis Gurney Smith, of Pennsylvania, was read and adopted. That on "Criminal Abortion," was referred to the Committee on Obstetrics. That on "Medical Education," was sent in printed by Dr. Geddings. That on "Prize Essays," by Dr. T. M. Logan, was read. The reports on the "Climatology and Epidemics," of various States, were for the most part, continued till next year. That on the "Climate, &c., of California," by Dr. F. W. Hatch, was referred to the Special Committee on the subject. A voluntary communication on "The Operations for Stone," was referred to the Committee on Surgery. After some discursive remarks by various members, the meeting was adjourned to 10, A. M., on Wednesday.

### SECOND DAY.

The Association met at 10, A. M., pursuant to adjournment. The attendance was large.

The minutes of Tuesday's session were read and approved.

The Committee of Arrangements and Credentials reported the names of accredited delegates and permanent members of the American Medical Association. The following members were present from the New England States:—

*Connecticut.*—E. R. Hunt, W. Woodruff, J. W. Phelps, Chas. L. Ives, Levi Ives, L. N. Beardsley, F. L. Diddle, W. B. DeForrest, B. H. Catlin, Alfred North, Moses C. White, Sheldon Beardsley, H. D. Holton, Henry McKnight.

*Massachusetts.*—George N. Thomson, H. R. Storer, E. Cutter, E. B. Moore.

*New Hampshire.*—John W. Parsons, J. L. Swett.

*Rhode Island.*—L. F. C. Garvin, G. L. Collins.

Dr. Ames, of Minnesota, moved that the report, with the exception of that portion referring to the members by invitation, be accepted.

Dr. Storer moved to amend the motion, in that the report be accepted as a whole, and not as at present adopted.

Dr. Toner desired to have the relations of Dr. Thomas, (of Philadelphia) to the Association defined.

Dr. Henry Gibbons doubted the propriety of catechizing members, after the Committee had accepted their names. It would establish a bad precedent, aside from creating unhealthy wrangles. He suggested the reference of the Thomas case to the Committee on Ethics—but he believed such a Committee did not exist.

Dr. Pinkney attempted to define his position, &c., but was declared out of order.

Dr. Pancks moved that the case of Dr. Thomas be referred to the Committee on Ethics; if none existed—holding over from last year—one might be appointed.

The President stated that Dr. Thomas was in full communion with the Association; no case for consideration existed.

Dr. Toner moved that the vote whereby the report of the committee on Credentials was accepted, be reconsidered.

Declared out of order.

Dr. Thomas arose to a question of privilege, and enumerated the Medical Societies in Philadelphia with which he was connected.

Dr. Storer remarked that Dr. T.'s explanation did not satisfy him. It showed that the gentleman was in better standing than he had supposed, but he favored the reference of the matter to the Committee on Ethics.

A delegate suggested that Dr. Pearson, of Woodland, occupied questionable relations with the Association.

Dr. Johnson, of Missouri, endorsed Dr. Pearson as a highly educated physician and able practitioner.

The Dr. Thomas case was finally referred to the Committee on Ethics by a vote of 85 to 15.

Dr. H. Gibbons stated that there was no Committee on Ethics in existence.

The President, by vote of the Association, was authorized to appoint a Committee on Ethics at an early day.

Dr. Logan presented a list of members of the San Francisco Medical Society, and moved that they be declared members of the Association by invitation.

Dr. Stout favored the motion, and recited cogent reasons for his action. California, situated on the verge of the continent, and yet in her infancy, failed to afford some of the facilities for progress fround in the East. Medical Societies were not numerous here, and chances for physicians to become elegible for membership to the National Society were comparatively few. It was for this reason that he supported the motion.

Dr. Simmons, as one of the Committee on Credentials, would have been pleased to recommend the gentlemen for membership, but found that the Constitution prohibited such action.

Dr. Davis, of Chicago, (Ill.), said that there were other medical gentlemen, outside of those read in the list by Dr. Logan, who were desirous of becoming members of the Association. The speaker did not favor excluding the gentlemen, by any means. Let them come in and witness our proceedings; extend cordial invitations to them to mingle with members of the Association; but they cannot be admitted as members. The Constitution would not permit the passage of the motion offered by Dr. Logan—and the Association must cling to the Constitution.

Dr. Logan's motion was lost, and a motion to invite the applicants to visit the meetings of the Association prevailed.

Dr. Yandell, of Kentucky, read a report of the Committee on Medical Education, prepared by Dr. Geddings, or South Carolina. In a private letter, Dr. Geddings notified the Association that the entire report was written by himself, without consulting other members of the committee.

On motion the report was accepted and referred to the Committee on publication.

In the discussion of the report, considerable time was occupied by appeals from the decision of the Chair, &c.

Dr. Henry Gibbons extended still farther invitations to the members, which were accepted.

Dr. Gibbons read an article on Vaccination, published in a homœopathic journal,* by Dr. Henry A. Martin, with his official title as Chairman of Committee on Vaccination of the American Medcal Association affixed. The opinions enunciated by the writer seemed to grate harshly on the ears of members of the profession. When he had finished reading the article, Dr. Gibbons moved for a reconsideration of the vote, whereby Dr. Martin was continued Chairman of the Committee on Vaccination for another year. The gentleman had insulted each and every member of the Association by the publication, and in justice to themselves immediate action should be taken in the matter.

Dr. Storer was unacquainted with the circumstances of the case,

---

* The New England Medical Gazette, January, 1871.

and felt that the Association should suspend judgment until Dr. Martin could be heard.

Members called for a second reading of the article.

Dr. Gibbons read the first few lines.

*Members*—" That's enough."

Dr. Dawson said that the article was an insult to every member of the Association, and moved that Dr. Martin be expelled as a member of the Association.

Dr. Bibb offered an amendment, that a committee of three be appointed to prefes charges against the gentleman.

Dr. Davis suggested the reference of the matter to the Massachusetts State Medical Society, to which Dr. Martin belonged.

Dr. Johnson gave Massachusetts a shot for her delinquencies; many of the members consorted with homœopathists in that State, hence nothing would be accomplished by referring the matter to the local Society there.

Dr. Stout offered an amendment to Dr. Bibb's motion—that the matter be referred to the Committee on Ethics.

Dr. Gibbons's motion to remove prevailed; Dr. Stout's amendment to refer the matter to the Committee on Ethics was also passed.

The Committee on Ethics was appointed by the Chair, and consists of Dr. Henry Gibbons, Dr. Davis, of Chicago, Dr. F. S. Smith, Dr. Parsons and Dr. Toner.

A motion to refer all questions of membership and character to the Committee on Ethics prevailed.

Several protests from Connecticut, Massachusetts and New York were referred to the Committee on Ethics.

Dr. T. M. Logan, of Sacramento, Chairman of the Committee on Prize Essays, reported in favor of awarding prizes as follows:—First prize—to E. R. Taylor, of Sacramento, for essay upon the "Chemical Constitution of the Bile." Second prize—to Benj. Howard, M. D., of New York, for essay upon "The direct method of artificial respiration for the treatment of persons apparently dead from suffocation, from drowning, or from other causes." Several other essays were received and considered.

On motion, the Committee on Prize Essays were instructed to return essays to writers when desired.

Dr. Davis of Chicago, member of the Committee on Resolutions, appointed at the meeting of the Association in 1869, submitted an elaborate report, closing with the following resolutions:

*Resolved*, That each State and local Medical Society be requested to provide, as a permanent part of its organization, a Board of Censors for determining the educational qualifications of such young men as propose to commence the study of medicine, and that no member of such Societies be permitted to receive a student into his office until such student presents a certificate of proper preliminary education from the Censors appointed for that purpose, or a degree from some literary college of known good standing.

*Resolved*, That a more complete organization of the profession in each State is greatly needed, for the purpose of affording a more efficient basis, both for educational and scientific purposes.

*Resolved,* That a committee of three be appointed for the purpose of continuing the correspondence with the State Medical Societies, and asking their earnest attention to the foregoing resolutions, in addition to those submitted for their action in 1869.

Dr. Moore, of St. Louis, offered a resolution that all medical colleges charge $100 as the fee for a course of lectures, and that a forfeiture of this rule shall exclude such college from representation in the Convention. After a protracted discussion, the resolution was voted down, on the ground that quality of education does not depend on price.

The resolutions offered were all tabled, and the Convention then adjourned until Thursday.

THIRD DAY.

The Association met pursuant to abjournment. In the absence of Dr. Stille, Dr. Henry Gibbons assumed the chair.

The Committee on Publication reported that the copy of Vol. XXI. was put into the hands of the printer on May 26th, 1870, but in consequence of ascertaining definitely, by means of circulars distributed to the members of the Association, how many copies it would be necessary or safe to print, the volume was not fairly started until the 1st of July. They then went to press, and 650 copies were printed. The report is accompanied by a table, exhibiting the number of copies of each volume, and the number disposed of since last report.

The Treasurer's report was read by the Secretary, from which we learn that the receipts during the year were $3,802.88; disbursements $3,098.56; the balance on hand is $704.32. The Treasurer reiterates the hope that the Association will not refer any matter to the Committee on Publication not of real value, as all matter thus referred must be published, at times causing the volume of transactions to cost more than the sum fixed for its purchase by the members.

Referred to the Committee on Publication.

The report of the Librarian, F. A. Ashford, M. D., of Washington, was received and read. He reported that the books entrusted to his custody by his predecessors had been well preserved at the Smithsonian Institute, through the kindness of Prof. Henry and its Regents. Three hundred and thirty-nine volumes, including pamphlets, monograms, &c., composed the collection at the date of the last report, and the additional matter received during the past year has been chiefly a continuation of the medical and surgical journals. The report is replete with important suggestions.

Referred to the Committee on Publication.

*Association of Superintendents of Insane Asylums.*—John C. Atlee, M. D., delegate to the Association of Medical Superintendents of American Institutions for the Insane, made a report, following which Dr. Storer offered the following resolution:

*Resolved*, That the Association of Superintendents of Institutions for the treatment of the Insane and the American Medical Association should be more closely united, and that the meetings of the two Associations should be held at about the same time and at the same place.

Adopted.

Dr. Johnson, of Missouri, presented a report from a special committee, suggesting a plan for the elevation of medical attainments and establishment of a National Academy of Medicine. Referred to Committee on Education.

Dr. Yandell, of the special committee, to whom was referred the report of Dr. Pinkney on Foreign Naval Medical Affairs, submitted at the session of the Association in 1870, presented the said report and moved its reference to the Committee on Education.

The motion prevailed.

Dr. E. T. Barber, of Yreka, submitted a report upon a case of fracture of the neck of the femur in a child seven years of age.

Referred to the committee on publication.

The Chairman of the Section on Materia Medica and Chemistry, Dr. Yandell reported having received a valuable paper from Dr. Gibbons, of Alameda, entitled *The Botany of the Pacific Coast.* The paper was accompanied by one hundred and eighty specimens of indigenous plants, &c., and would certainly be considered a valuable contribution to the science of medicine.

The committee moved that the paper be referred to the committee on publication.

Dr. Gibbons arose and requested that the recommendation of the committee be withdrawn. The paper was not complete—not as perfect as he could make it by additional work.

On motion, a vote of thanks was passed, and the paper returned to its author for completion.

Dr. H. R. Storer, delegate from the American Medical Association to the Canadian Medical Association, submitted a verbal report in behalf of himeelf and associates—Dr. Sullivan, of Boston, and Dr. Gerrish of New York. He eulogized the Canadian Association. Its members were far above the members of the American Association in point of medical education—almost all of them having graduated from European Colleges of note.

The committee on Nominations made the following report: For President, Dr. D. W. Yandall, of Kentucky; First Vice-President, Thos. M. Logan, of California; Second Vice-President, C. L. Ives, of Alabama; Third Vice-President, R. M. Mitchell, of Alabama; Fourth Vice-President, J. K. Bartlett, of Wisconsin; Assistant Secretary, D. Murray Chester; Librarian, F. A. Ashford, Philadelphia; Treasurer, C. Weston, Philadelphia. Next place of meeting, Philadelphia.

On motion of Dr. Davis, the report was accepted, and the officers unanimously elected.

The Committee on Ethics submitted a partial report, recommending some removals, &c., and asking time in the case of Dr.

Thomas, the delegate from the Female College of Philadelphia.

The report was accepted.

Under the head of unfinished business, an amendment to the Constitution, offered at the last meeting of the Association by Dr. Hartshorne, of Philadelphia, was taken up for consideration. The proposed amendment is embodied in the following resolution :—

"*Resolved,* That nothing in this Constitution shall be so construed as to prevent delegates from colleges in which women are taught and graduated in medicine, and hospitals in which medical women, graduates in medicine, attend, from being received as members of this Association.

A lively discussion ensued, in the course of which remarks were made in favor of the resolution by Drs. Harding of Indiana, King of Pennsylvania, Gibbons of California, Atlee of Pennsylvania, and Thomas of Pennsylvania; and in opposition by Drs. Davis of Illinois, Johnson of Missouri, and McArthur of Illinois. A vote was taken, and the motion to adopt the resolution was indefinitely postponed. The Convention then adjourned until Friday.

FOURTH DAY.

The Association assembled at 9, A. M., President Stille in the Chair.

A number of the delegates having departed for the interior, the attendance did not equal that of previous sessions.

The minutes of preceding meetings were read and approved.

Dr. T. M. Logan, of Sacramento, submitted a series of resolutions recommending the establishment of a chair of hygiene in medical schools, and suggesting a National Health Council based on the principle of the State Boards of Health of Massachusetts and California.

Adopted, and referred to the Committee on Publication.

Dr. Logan moved that the State of Pennsylvania be represented by the President, Dr. Stille, on the proposed Committee. Carried.

The nominating committee reported the names of gentlemen selected by them for the various standing committees and for the officers of the sections.

The Secretary read the minutes of the Committee on Obstetrics and Medical Jurisprudence. Referred to the Committee on Publication.

Dr. O'Donnell offered a resolution condemning criminal abortion, and urging stringent measures for its prevention.

Surgeon J. M. Brown, of the United States Navy, returned the thanks of the medical gentlemen of this department of the public service for the hearty cooperation of the Association in the recent contest between line and staff; a contest to define the position and rights of the latter, and acknowledge the dignity of the profession.

The law now recognized the usefulness of the staff, and regulated the rank of officers; it did not give them all they were entitled to, but enough on which to make an honorable concession and a fair compromise.

Referred to the Committee on Publication.

Dr. Montgomery, of Sacramento, offered a resolution to the effect that a Chair of Ethics should be established in all the Medical Colleges in the United States, either as an Independent Chair or in connection with some other department. Withdrawn.

The number of licensed physicians in the United States has been ascertained by Dr. J. M. Toner, after considerable labor—according to the statement of Dr. McArthur, of Illinois. There are some 60,000 physicians; only 3,000 of them homœopaths. In view of the importance of these statistics, it was moved that they be referred to the Committee on Publication.

The motion prevailed.

In view of the fact that a proposition for a memorial to Sir James Y. Simpson had been inaugurated by the physicians of Europe and Canada, and that the co-operation of the American Medcial Association was desired, Dr. Storer moved that the Association take the necessary steps in the matter as an evidence of their appreciation of the deceased. Carried.

The Committee on Ethics reported to refer the case of Dr. Martin of Massachusetts, mentioned in the record of the first day's meeting, to the local society. Dr. T. M. Wise of Kentucky, was appointed Chairman of the Committee on Vaccination, in place of Dr. Martin, removed.

Dr. Atlee, of Philadelphia, offered the following resolution:—

*Resolved*, That the American Medical Association acknowledges the right of its members to meet in consultation the graduates and teachers of Women's Medical Colleges, provided the code of ethics of the Association is observed.

Dr. Storer hoped that no action would be taken on the resolution. Inasmuch as the question was discussed fully yesterday, he would protest against the question coming up again. He thought that the sense of the Association was fully ascertained by the votes already taken.

Dr. Johnson, of Missouri, had a few words to say in behalf of the resolution. He hoped it would pass. This was not a question as to the admission of women into the Association; it was merely a resolution to protect the medical science. He would regret to have the women assailed by the Association; any honorable man would agree with him on that proposition. Let the women have their own associations and manage their own affairs—but when it comes to consulting, all barriers should be removed.

A sprightly discussion then ensued, which was engaged in by various members of the Convention; the proceedings assumed an uproarious character, and an incessant din took the place of legitimate debate.

The question recurred upon the original resolution.

Dr. J. M. Brown moved that the subject matter be indefinitely postponed.

Dr. Toner moved to lay the resolution upon the table.

The President called for an expression of opinion by the Association.

Misunderstanding the question before the house, many delegates arose, then became seated, and continued to give evidence of indecision, until the body of the house recalled reminiscences of the fishing excursion by the incessant bobbing in progress.

Finally a delegate called upon the President to state the question.

Dr. Atlee called for a vote upon his original proposition.

Dr. Davis desired to know if the Association would falsify its record of yesterday, and continue to wrangle until it was too late to go over the bay. The question under consideration did not amount to any more than tweedledee and tweedledum at best.

*Dr. Cole*—I move that we adjourn until 8 o'clock this evening, and make the consideration of this resolution the special order. Carried.

The members of the Association, together with other invited guests, proceeded on an excersion to Oakland.

EVENING SESSION.

The Association assembled in the evening, pursuant to adjournment, President Stille in the chair.

The resolution on the female physician, the special order of the evening, was discussed with great freedom. Finally after a spicy debate—

Dr. Matherly suggested that the American Medical Association had no authority for meddling in local quarrels, and therefore moved an indefinite postponement of the subject matter.

The motion prevailed.

Dr. Storer submitted the following resolution:—

*Resolved*, That this Association views with dissatisfaction the course of gentlemen who, in setting at defiance their local and State Societies, have contemplated the establishment of a precedent that, admitted in other matters, would at once destroy the authority of this Association.

Indefinitely postponed, on motion of Dr. Gibbons.

Resolutions of thanks to the officers, the Press, and Railroad Companies, were passed, after which the meeting adjourned *sine die*. *Boston Medical and Surgical Journal.*

